# First Evidence of the Toxin Domoic Acid in Antarctic Diatom Species

**DOI:** 10.3390/toxins13020093

**Published:** 2021-01-26

**Authors:** Anna J. Olesen, Anneliese Leithoff, Andreas Altenburger, Bernd Krock, Bánk Beszteri, Sarah Lena Eggers, Nina Lundholm

**Affiliations:** 1 Natural History Museum of Denmark, University of Copenhagen, Øster Farimagsgade 5, 1353 Copenhagen K, Denmark; alc@science.ku.dk (A.L.); nlundholm@snm.ku.dk (N.L.); 2 The Arctic University Museum of Norway, UiT The Arctic University of Norway, Lars Thørings veg 10, 9006 Tromsø, Norway; andreas.altenburger@uit.no; 3 Ökologische Chemie, Alfred Wegener Institut-Helmholtz Zentrum für Polar-und Meeresforschung, Am Handelshafen 12, 27570 Bremerhaven, Germany; bernd.krock@awi.de (B.K.); lena.eggers@awi.de (S.L.E.); 4 Department of Phycology, Faculty of Biology, University of Duisburg-Essen, Universitätsstrasse 2, 45141 Essen, Germany; bank.beszteri@uni-due.de

**Keywords:** Antarctic, Domoic Acid, Iso-Domoic acid, HAB, Southern Ocean

## Abstract

The Southern Ocean is one of the most productive ecosystems in the world. It is an area heavily dependent on marine primary production and serving as a feeding ground for numerous seabirds and marine mammals. Therefore, the phytoplankton composition and presence of toxic species are of crucial importance. Fifteen monoclonal strains of *Pseudo-nitzschia subcurvata*, a diatom species endemic to the Southern Ocean, were established, which were characterized by morphological and molecular data and then analysed for toxin content. The neurotoxins domoic acid and iso-domoic acid C were present in three of the strains, which is a finding that represents the first evidence of these toxins in strains from Antarctic waters. Toxic phytoplankton in Antarctic waters are still largely unexplored, and their effects on the ecosystem are not well understood. Considering *P. subcurvata*’s prevalence throughout the Southern Ocean, these results highlight the need for further investigations of the harmful properties on the Antarctic phytoplankton community as well as the presence of the toxins in the Antarctic food web, especially in the light of a changing climate.

## 1. Introduction

The unique Antarctic marine ecosystem is fueled by phytoplankton, particularly diatoms, capturing energy from the sun. The potentially toxic diatom genus *Pseudo-nitzschia* is among the most frequently encountered and dominant diatom genera in Antarctic waters, e.g., contributing 13–70% of diatom densities in the Weddell Sea [1,2]. Despite this, nothing is known about the toxicity of the genus in Antarctic waters [2,3], whereas *Pseudo-nitzschia* is a known producer of the neurotoxin domoic acid (DA) in temperate and tropical waters, causing amnesic shellfish poisoning in humans [1]. DA accumulates in a wide range of planktonic and benthic organisms across the marine food web, such as krill, copepods, fish, and bivalves [1]. In marine mammals, this can e.g., result in acute and chronic poisoning, with effects such as reduced reproduction, seizures, and death [4]. Worldwide monitoring efforts have linked several toxic *Pseudo-nitzschia* blooms to unusual, large-scale mortality events in a range of marine vertebrates including sea lions, whales, and seabirds [4,5,6]. However, the term “worldwide monitoring” does not include the polar regions, which are areas characterized by poorly known phytoplankton diversity and the absence of regular phytoplankton monitoring. Presently, 27 species of *Pseudo-nitzschia* can potentially produce DA [1,7,8,9]. In addition to DA, eight different DA isomers (A–H) exist, of which three (DA-IA, DA-IB, and DA-IC) are found in *Pseudo-nitzschia* [1,10,11].

The presently known *Pseudo-nitzschia* diversity in Antarctic waters is relatively low, which is based only on a restricted number of studies on morphological diversity. However, a high richness of unique rDNA gene sequences suggest a considerably larger *Pseudo-nitzschia* diversity [3]. Six different species have presently been recorded from Antarctic waters: three endemic for Antarctica (*P. prolongatoides, P. subcurvata,* and *P. turgiduloides*) while the remaining, *P. lineola, P. heimii,* and *P. turgidula,* are also found in other regions [1,2,12,13,14]. Until now, the few Antarctic strains studied (*P*. *subcurvata, P. turgiduloides,* and *P. lineola*) have shown no sign of DA production [12,13,14].

Nothing is known about the fate of the toxins in the Antarctic food web. The marine habitat of the Arctic is comparable to that of Antarctica. In the Arctic region, DA has been found in the gut and feces of all 13 different Alaskan marine mammal species examined and at levels high enough to have an impact on mammal health [5]. The lack of knowledge of toxic phytoplankton and toxin impact on the marine food web in Antarctica illustrates the inaccessibility of the region, not the relevance. Globally, harmful algal blooms are getting more frequent, which is probably linked to climate change [15]. If *Pseudo-nitzschia* species from Antarctica can produce DA, impacts on higher food web levels must be expected.

During two different cruises in Antarctic waters, several strains of *Pseudo-nitzschia* were established as clonal cultures, which were characterized using both morphological (TEM and SEM) and molecular data (Internal transcribed spacers (ITS) of ribosomal DNA (rDNA)) and studied for cellular DA content.

## 2. Results

**Figure 1:**
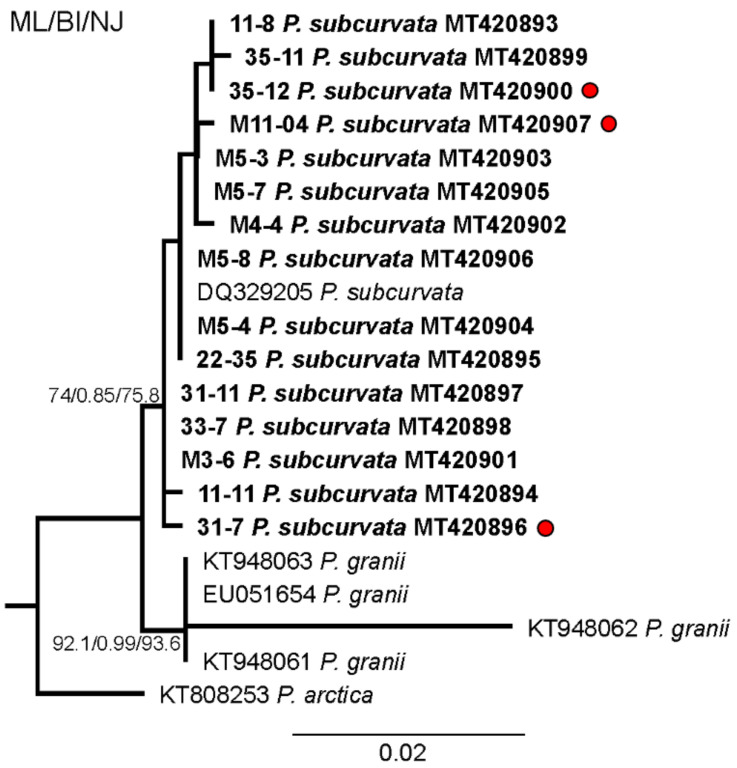
Phylogenetic analysis based on maximum likelihood (ML) of the strains of *P. subcurvata* included in this study. Numbers at nodes represent the bootstrap values of an ML analysis with 1000 replicates/posterior probability of Bayesian inference (BI) analysis/bootstrap values of neighbour-joining (NJ) out of 10,000 replicates. The scale bar corresponds to two substitutions per 100 nucleotide positions.

**Figure 2:**
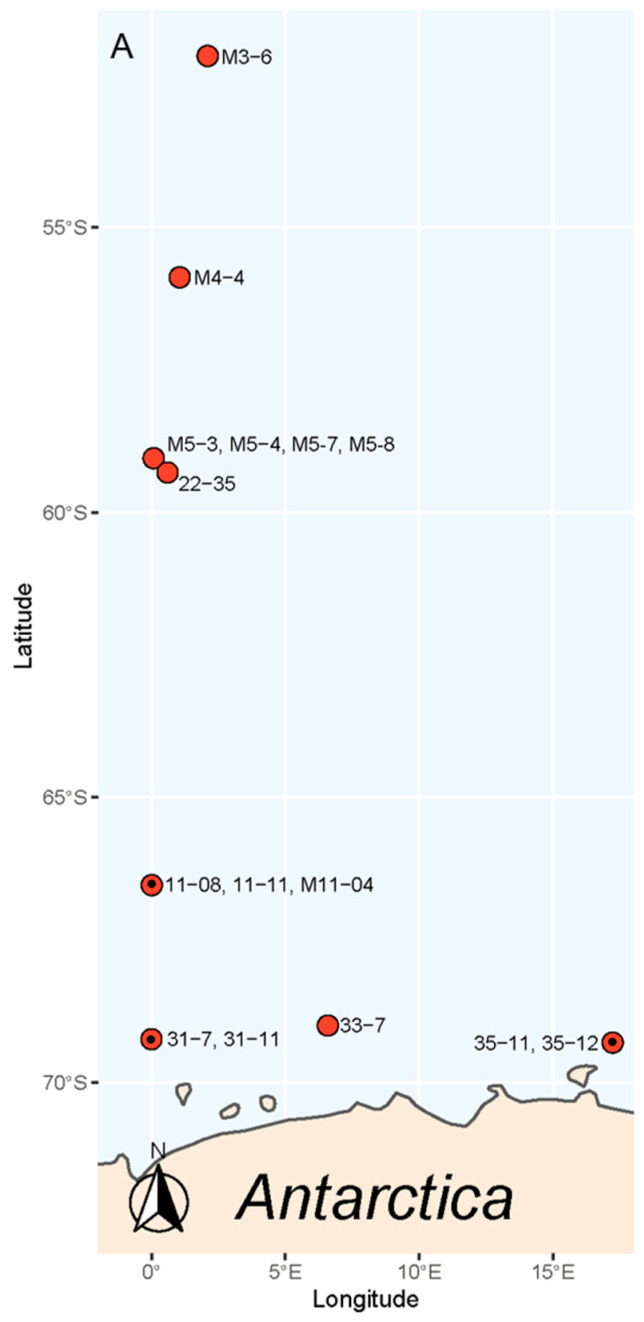
Map of the area showing where each of the strains of *P. subcurvata* were isolated, stations marked with a dot (**·**) contained toxins. The strain name is given by the station.

In total, 15 monoclonal strains of *P. subcurvata* (Figure 1) were isolated and established in culture from samples originating from eight different stations during two separate sampling cruises in the Southern Ocean (Figure 2). Water temperatures varied between −1.58 and 2.46 °C, and salinities were stable, on average 33.98 ± 0.16. Silicate and nitrate peaked at station 31, close to the Antarctic mainland with levels of approximately 120 µM for both nutrients (Supplementary Table S1). Silicate levels were otherwise ranging between 25.95 and 66.72 µM, which were levels that were assumingly not limiting for diatom growth. Nitrate was found in relative high concentrations, except at stations 3, 4, and 5, where it was found in low levels (0.08–0.24 µM). Phosphate levels were stable around 1.61 µM ± 0.67, which are levels possibly limiting for growth in areas with high nitrogen levels.

**Figure 3:**
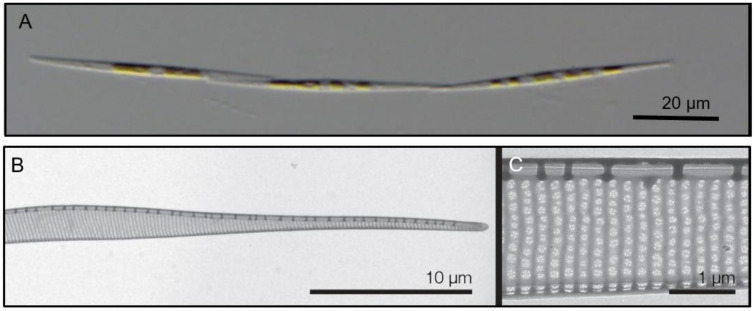
(**A**) Light micrograph of a chain of *P. subcurvata.* (**B**,**C**) TEM micrographs of a *P. subcurvata* valve (M11-04) showing half part of diatom frustule, lack of central nodule, interstriae and fibulae, and one row of poroids with detailed poroid structure.

The monoclonal strains were identified based on qualitative and quantitative analyses of transmission electron micrographs of frustule structures (Figure 3). The analyses showed the subcurvate shape of cells, the absence of a central nodule, a fibula density of 12–20 in 10 µm, and a stria density of 40–50 in 10 µm. In combination with the presence of one row of poroids, with each poroid comprising four to eight sectors, morphological characters were in agreement with descriptions of *P. subcurvata* [16]. The phylogenetic analyses of ITS rDNA data showed the *P. subcurvata* strains to cluster in a clade comprising *P. subcurvata* from Genbank, with *P. granii* as a sister clade (Figure 1). Combined results from transmission electron microscopy and from ITS rDNA phylogenetic analyses revealed the identity of all 15 strains being *P. subcurvata*, with very limited variation in morphology and in their ITS rDNA data.

**Figure 4:**
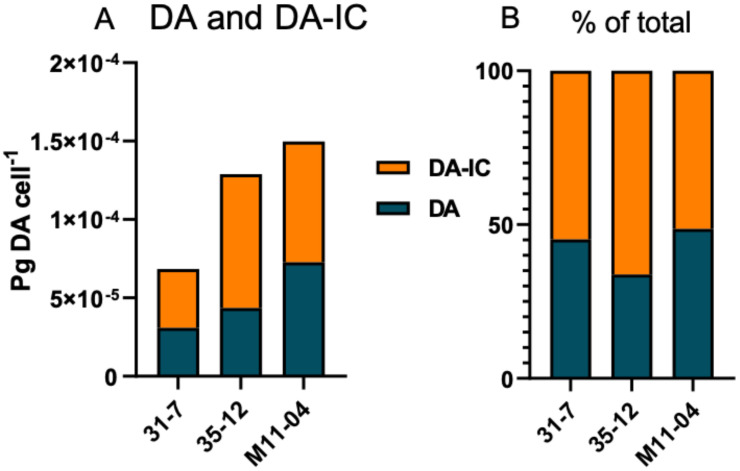
(**A**) Domoic acid (DA) and iso-domoic acid C (DA-IC) profiles of the three toxic strains, absolute amounts. (**B**) Relative amounts of DA and DA-IC of the three toxic strains.

Toxin analyses of all 15 strains sampled in the early stationary growth phase revealed the presence of DA and iso-domoic acid C (DA-IC) in three of the 15 *P. subcurvata* strains (31-7, 35-12, and M11-04) (Figure 2 and Figure 4, and Table 1). The toxic strains were collected at stations 31, 35, and 11 (Figure 2 and Supplementary Table S1), which are all stations close to the Antarctic mainland. Five non-toxic strains were also isolated from the same area close to the Antarctic mainland. Further away from the Antarctic mainland (Figure 2), all seven strains were found to be non-toxic or with toxins at levels below the detection level.

**Figure 5:**
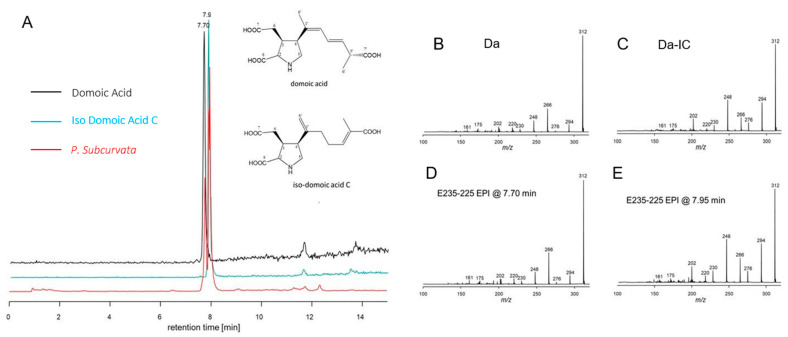
(**A**) Extracted ion chromatograms (*m/z* 312 > 266) of domoic acid (black), iso-domoic acid C (green), and of compounds found in strain M11-04 (red) and structures of DA and DA-IC. (**B**) Collision-induced dissociation (CID) spectrum of DA (**C**) CID spectrum of DA-IC, (**D**) CID spectrum of compound *m/z* 312 eluting at 7.70 min of strain M11-04, and (**E**) CID spectrum of compound *m/z* 312 eluting at 7.95 min of strain M11-04.

The total cellular DA amount ranged from 6.85 × 10^−5^ to 1.5 × 10^−4^ pg DA cell^-1^ (Figure 4, Table 1). Apart from DA, an isobaric compound was registered. Collision-induced dissociation (CID) spectra of DA and the isobaric compound differed only with differences between fragment intensities (Figure 5). The high similarity of the CID spectra of DA and the compound led to the hypothesis that the compound was an isomer of DA. This hypothesis was verified by an analytical standard of DA-IC, which was received from Pearse Mc Carron of the NRC-IMB in Halifax, NS, Canada, showing the same retention time and CID spectrum as the compound from the toxic *P. subcurvata* strains (Figure 5). DA-IC was found in all three strains, in amounts ranging from 3.76 × 10^−5^ to 8.54 × 10^−5^ pg DA-IC cell^-1^ (Figure 4). Domoic acid accounted for approximately half of the total DA content in all three strains, with relative amounts ranging from 51% to 66% (Figure 4B). When comparing the cellular content of total-DA (DA+DA-IC), strain 31-7 contained less than 35-12 and M11-04 (*p* < 0.05), but no statistical testing was possible due the lack of replicate DA samples.

Cell volumes of the three toxic strains varied from 162.5 to 235.8 µm^3^. Taking differences in cell volume into consideration, the toxin levels per cell volume showed M11-04 and 35-12 as being more potent toxin producers pr. cell volume. Total toxin content varied from 2.9 × 10^−7^ total DA pr. µm^3^ in 31-7 and peaked in M11-04 with 8.7 × 10^−7^ total DA pr. µm^3^.

## 3. Discussion

The finding of both DA and DA-IC in three Antarctic strains of *P. subcurvata* represents the first evidence of these toxins in Antarctic phytoplankton strains (Figure 4, Table 1). The presence of three toxic and twelve non-toxic strains of *P. subcurvata* explains the previous records of Antarctic *Pseudo-nitzschia* species as non-toxic [14,17]. Until now, only a total of seven strains of *Pseudo-nitzschia* species from Antarctic waters have been examined for DA content, i.e., four strains of *P. subcurvata*, two strains of *P. turgiduloides*, and one strain of *P. lineola* [14,17]. Hence, the present study triples the total number of *Pseudo-nitzschia* strains studied, and furthermore, detection levels might be lower in present-day studies than in studies published in early 1990s. As we found DA in three of 15 strains, the results revealed high intraspecific variation in total DA content and high likelihood of isolating and analysing non-toxic strains of possibly toxic species. These results support previous findings of high intraspecific diversity in toxin levels in other *Pseudo-nitzschia* species [1]. The time range covered by the sampling of the toxic strains (Supplementary Table S1) indicates the presence of toxic strains as frequent components of the Antarctic phytoplankton community. The strain containing most toxin (M11-04) is the oldest strain; however, the amounts are low in all strains but relatively comparable to other small toxic *Pseudo-nitzschia* species (Table 2), even though comparisons should be made with caution, as the species might be cultured under different conditions. The three toxic strains were all isolated close to the Antarctic mainland, along with five non-toxic strains, whereas all seven strains isolated farther away from the mainland were non-toxic, which is a finding that could suggest a difference in toxicity between populations of *P. subcurvata* but could simply also be a coincidence due to the relatively small number of strains examined.

Our evidence of DA and DA-IC in Antarctic phytoplankton supports previous findings of DA being present in the water column of the Southern Ocean during a large-scale iron fertilizer experiment in 2002 in a concentration of up to 220 ng L^−1^ [12]. They linked the presence of DA to the *Pseudo-nitzschia* species *P. granii*, as it was one of four and the dominant *Pseudo-nitzschia* species present during the experiment. *P. subcurvata* was not present in the water samples during the time of the experiment. Adding the finding of toxic *P. subcurvata* to the extremely limited number of studies in this area, as well as the report of DA in the water column coinciding with the presence of *P. granii*, it is reasonable to suggest that more toxigenic *Pseudo-nitzschia* species might be present in the Southern Ocean. Calculations suggest that *P. granii* contained orders of magnitude (0.85 pg cell^−1^) more toxin during the oceanic iron fertilizer experiment than *P. subcurvata* from the current investigation, indicating that some *Pseudo-nitzschia* species in the Southern Ocean can attain higher DA contents depending on the environmental conditions (see below). The toxic content in the strains included in the current study were low but comparable to other strains in non-inducing conditions; see Table 1 [1]. *P. granii* and *P. subcurvata* are genetically very similar, and they cluster together in the phylogenetic analysis (Figure 1) [8]. The *P. granii* cells analyzed during the iron-fertilizer experiment were under toxin-inducing conditions, with high iron concentrations, possibly grazer presence, and in competition with other phytoplankton species [1,12]. Taking these inducing conditions or the absence of them into consideration, it is possible that *P. subcurvata* comprises similar toxic potential as *P. granii*. Different chemical, physical, and biological factors can affect the toxicity of *Pseudo-nitzschia* cells, inducing higher toxicity e.g., under the depletion of silicate and phosphate and the presence of herbivorous grazers [1,11,15,28]. Therefore, future studies exploring the toxigenic potential of Antarctic *Pseudo-nitzschia* strains could be relevant.

The current study is consistent with a large-scale study in the transect of the east Atlantic Ocean, detecting dissolved DA in the Southern Ocean [29]. The highest dissolved DA concentration was found around the equator; dissolved DA was detectable in all surface water samples as south as 70° S [29]. This knowledge adds to the pivotal importance of studying DA presence and impact in global as well as Antactic ecosystems.

The present study is the second report of DA-IC in diatoms (Figure 5, Table 1), the first study was on *P. australis* isolated close to New Zealand, and the exact cellular amount is not described [10,30]. In the present study, levels of DA-IC exceeded the DA level in all three strains (Figure 4). However, previous studies on a subtropical diatom of another genus, *Nitzschia navis-varingica*, showed similar high relative amounts of isomers. In one study, DA-IA and DA-IB were found in concentrations of approximately 50% of the total DA content [31]. The limited knowledge on isomers of domoic acid is most likely reflecting a monitoring focus on DA and therefore limited focus on the presence of isomers, because DA is regulated in seafood production and because we know very little about the isomers. Isomers of DA are not regulated globally in industrial seafood production. The toxic potential of DA-IC is debated; one study suggested it to be one-third of DA [11], whereas another study on isomers from *P. australis* assessed the binding affinity for DA-IC compared to DA and found it 240-fold less potent. A third study found DA-IC to be 20 times less potent than DA [32]. Conclusively, more studies are needed to elucidate the true toxic potential of DA-IC.

Our findings support previous detections of DA in water samples in the Southern Ocean [12]. *P. subcurvata* is an endemic, widely distributed, and frequent component of the marine ecosystem in Antarctica [2]. Other *Pseudo-nitzschia* species have been recorded and are abundant in Antarctic waters, illustrating the relevance of expanding studies to more *Pseudo-nitzschia* species, which could also comprise both toxic and non-toxic clones. The temperature optimum for the Antarctic *P. subcurvata* is interestingly around 8 ℃, which is well above the present temperatures of the area [33]. As global temperatures are increasing, toxic *P. subcurvata* will, according to a study, enhance its competitive advantage and thus represent an increasing threat for Antarctic fauna [33]. The toxicity and frequency of harmful algae blooms are often hypothesized as increasing with global ocean warming [33]. Other factors can also enhance the toxic potential of the *Pseudo-nitzschia* community in the Antarctic region, like variation in nutrient availability, presence of toxin-inducing grazers (e.g., krill and copepods), variation in salinity, and pH of the ocean. These are factors known to affect the toxic potential of *Pseudo-nitzschia* communities, and the combined effect of these factors is largely unexplored [1] and completely unknown in the context of Antarctic species.

The present study shows, for the first time, DA presence in *Pseudo-nitzschia* strains from Antarctica. The Antarctic Southern Ocean is one of the most productive ecosystems in the world. The area is serving as a feeding ground for many seabirds and marine mammals. The area is especially important to the humpback whale (*Megaptera novaeangliae*) and the southern right whale (*Eubalena australis*). Both are dependent on the Antarctic region to feed on krill before the long fasting periods, which they spend calving at lower latitudes [34,35]. However, krill is an important vector for DA [15], and DA can cross the placental barrier of pregnant marine mammals [15]. This raises concern for the accumulation of DA in marine mammals in the Antarctic region [4,34,35]. Thus, toxigenic blooms of DA-producing diatoms have the potential to disturb a marine ecosystem, which is already susceptible to the effects of climate change. Thus, the effort of further mapping and exploring DA and *Pseudo-nitzschia* species in the Southern Ocean is of major importance.

## 4. Materials and Methods

Water samples and physiochemical parameters were collected with a conductivity, temperature, depth sampler (CTD) at depths of 10–15 m (Supplementary Table S1). The samples were collected between 51.99° S as the most northern and 69.3° S as the southern locality. Strains were isolated from water sampled at different localities (Figure 2, Supplementary Table S1) close to the Antarctic mainland. Monoclonal strains were established by isolation of single cells or chains. Strains were cultured in 55 mL flasks in L1 medium with salinity 30 at 4 °C and 120-µmol photons m^−2^ s^−1^ cool white light.

Cultures were harvested for toxin analyses at a density of 350,000–750,000 cells mL^−1^ in early stationary phase. All cultures were harvested for toxin analysis in April 2019; for this reason, some cultures were approximately 1.5 years and others were approximately 4 months (see Supplementary Table S1). A 40–45 mL well-mixed sample from each culture was centrifuged at 4 °C, 1811× *g* for 15 min, and the cell pellet was pooled and centrifuged once again at 1811× *g* for 15 min, the supernatant was the removed, and the pellet was stored at −20 °C until further analysis. Nutrient analysis was done using a QuAAtro Seal AutoAnalyzer following standard colorimetric techniques. The accuracy of the analysis was evaluated via the measurement of KANSO LTD Japan Certified Reference Materials, and corrections were applied if required.

For toxin analysis, 300–400 µL extraction solvent was added to the harvested pellet, vortexed, and solution transferred to cryotubes. Extraction was carried out following [36] and transferred to HPLC glass vials, sealed, and frozen until analysis. DA contents were measured using liquid chromatography coupled with tandem mass spectrometry (LC-MS-MS) [36].

Strains were identified as *P. subcurvata* by morphological studies (Figure 3) and molecular analyses of the ITS rDNA regions (ITS1, 5.8S and ITS2 ribosomal DNA) (Figure 1). DNA was extracted from cell pellets using the CTAB method [37]. For amplification of ITS rDNA, the primer pair used was forward: ITS1 (5′-TCCGTAGGTGAACCTGCGG-3′) and reverse: ITS4 (5′-TCCTCCGCTTATTGATATGC-3′) or the newly designed ITS4Ps (5′-TCCTCCGCTTAATTATATGC-3′). PCR reactions were done in 25 µl reactions containing 1.5 mM MgCl2, 0.8 mM dNTPs (deoxyribose adenosine triphosphate) [VWR #733-1363], 0.5 units’ polymerase [VWR #733-1301], and 0.4 µM primers using 36 cycles and 55 °C as the annealing temperature. The PCR products were sent to Macrogen (Macrogen Europe, Amsterdam, NL) for purification and sequencing in both directions. Sequence analysis (trimming, assembly, BLAST) was done with Geneious version 2020.0.3 (Biomatters Ltd., Auckland, New Zealand).

Additional sequences of ITS rDNA were downloaded from GenBank and aligned using MAFFT with subsequent alignment masking, as implemented in GUIDANCE2 (34). The GUIDANCE alignment score was 0.99. The masked alignment (columns below confidence score of 0.93 were removed) was trimmed by hand and included 724 characters. The alignment was uploaded to the ATGC bioinformatics platform for PhyML 3.0 analysis with Smart Model Selection (best model was HKY85+I), using the Akaike Information Criterion and performing 1000 bootstrap replicates. Bayesian Inference was performed with MrBayes 3.2.6 using the HKY85+I model as implemented in Geneious^®^ 2020.1.2. The following settings were used: four simultaneous Markov chain Monte Carlo (MCMC) run for 1,000,000 generations, sampling every 1000 generations. The first 25% of trees were discarded as burn-in. Finally, a neighbor-joining tree was built, using the HKY model and 10,000 bootstrap replicates as implemented in Geneious Prime^®^ 2020.1.2.

Cultures were rinsed for morphological analyses during March and April 2019, leaving some cultures 1.5 years old and others approximately 4 months old; the method used was [37]: a drop of the material was mounted on carbon-coated grids and left to dry. The grids were inspected in a transmission electron microscope (TEM) (JEOL 1010, Tokyo, Japan). For SEM (JEOL JSM 6335F, Tokyo, Japan), a few drops of rinsed material were mounted on round cover glasses and glued to metal stubs with double-sticky tape. The stubs were dried, sputter-coated with gold/palladium, and examined.

A minimum of three different valves from each culture was measured and included in the species determination.

Cell volume calculation: Volume = (0.6 × L × W^2^) + (0.4 × 0.5 × L × W^2^).

Where L is the cell length and W is the width of the cell.

## Figures and Tables

**Table 1:** Table overview of the toxin content, limit of detection, cell number in the DA sample pellet, and cell volume of the three toxin-containing strains.

ID	pg. DA cell^−1^	pg. DA-IC cell^−1^	Limit of Detection (pg cell^−1^)	Cells in DA pellet	Cell Volume (µm^3^)
**31-7**	3.09 × 10^−5^	3.76 × 10^−5^	1.38 × 10^−5^	32.5 × 10^6^	235.8
**35-12**	4.36 × 10^−5^	8.54 × 10^−5^	2.75 × 10^−5^	16.4 × 10^2^	162.5
**M11-04**	7.28 × 10^−5^	7.69 × 10^−5^	1.74 × 10^−5^	28.8 × 10^6^	171.7

**Table 2:** Comparative species to *P. subcurvata* and their levels of toxins measured in laboratory (max and min if available). The species are selected based on the criteria: DA assessments without induction factors and small species.

Species	Total pg DA cell^−1^	Location	Reference
***P. turgidula***	Max: 3.3 × 10^−2^ Min: 0.52 × 10^−5^	Tauranga Harbor, New Zealand Ocean Station PAPA (NE Pacific)	[18,19]
***P. cuspidata***	Max: 3.1 × 10^−2^ Min: 1.9 × 10^−2^	Washington State coastal waters	[20,21]
***P. delicatissima***	Min: 0.2 × 10^−3^ Max: 0.5 × 10^−2^	Prince Edward Island, Canada	[22,23]
***P. pseudodelicatissima***	0.78 × 10^−2^	Thermaikos Gulf, Greece	[24]
***P. calliantha***	Max: 4.3 × 10^−1^ Min: 0.57 × 10^−2^	Black Sea Chesapeake Bay, Maryland, USA	[25,26]
***P. galaxiae***	0.36 × 10^−3^	Gulf of Naples, Italy	[27]
***P. subcurvata***	Min. 6.85 × 10^−5^ max: 1.5 × 10^−4^	Southern Ocean	Current study

## Data Availability

Data available in a publicly accessible repository that does not issue DOIs. Publicly available datasets were analyzed in this study. This data can be found here: [Figure 1 GenBank Accession numbers].

## Supplementary Materials

The following are available online at https://www.mdpi.com/2072-6651/13/2/93/s1, Table S1: Detailed overview of the strains included in this study. Date of isolation, location for specific water sample, temperature, salinity and nutrient levels when available.
