# Supplementary material for: First Evidence of the Toxin Domoic Acid in Antarctic Diatom Species
Source: Toxins (Basel). 2021 Jan 26;13(2):93. doi: 10.3390/toxins13020093 (PMC7912347; doi:10.3390/toxins13020093)
Supplement: Supplementary file 1 [file toxins-13-00093-s001.pdf]

Supplementary Materials: First Evidence of the Toxin Domoic Acid in Antarctic Diatom Species

Anna J. Olesen, Anneliese Leithoff, Andreas Altenburger, Bernd Krock, Bánk Beszteri, Sarah Lena Eggers and Nina Lundholm

Table S1. Detailed overview of the strains included in this study. Date of isolation, location for specific water sample, temperature, salinity and nutrient levels when available.

Station	Strain ID	isolation date	Lat.	Long.	Temp. (°C)	Salinity	Si (μmol L^{-1})	PO_4 (μmol L^{-1})	NO_3 (μmol L^{-1})
**3**	M3-6	26/12/2016	51.99°S	2.10°E	2.46	33.77	25.95	1.58	0.24
**4**	M4-4	26/12/2016	55.88°S	1.06°E	1.7	33.79	52.88	1.22	0.08
**5**	M5-3	27/12/2016	59.05°S	0.08°E	N/A	N/A	55.89	1.14	0.13
	M5-4	27/12/2016							
	M5-7	30/12/2016							
	M5-8	30/12/2016							
**11**	M11-04•	28/12/2016							
	11-8	28/12/2016	66.54°S	0.01°E	2.86	34.14			
	11-11	28/12/2016							
**22**	22-35	31/12/2018	59.3°S	0.6°E	−0.33	33.98	58.25	1.62	24.63
**31**	31-7•	04/01/2019	69.24°S	0.0°	−1.58	34.10	120.65	2.35	120.65
	31-11	04/01/2019							
**33**	33-7	05/01/2019	69°S	6.59°E					
**35**	35-11	08/01/2019	69.3°S	17.23°E	-1.35	34.14	66.72	1.75	25.97
	35-12•	08/01/2019							

Strains marked with • and orange contained DA and Iso-DA C.
